# Lessons Learnt from Epidemiological Investigation of Lassa Fever Outbreak in a Southwest State of Nigeria December 2015 to April 2016

**DOI:** 10.1371/currents.outbreaks.bc4396a6650d0ed1985d731583bf5ded

**Published:** 2018-06-29

**Authors:** Elvis Efe Isere, Akinola Ayoola Fatiregun, Olayinka Ilesanmi, Ibidolapo Ijarotimi, Beatrice Egube, Adewale Adejugbagbe, Gboyega Adekunle Famokun

**Affiliations:** World Health Organization; WCO, NigeriaWorld Health Organization; Federal Medical Centre; State Specialist Hospital, Akure; State Specialist Hospital; Ondo State Primary Health Care Development Board, Ondo State, Nigeria; State Ministry of Health

## Abstract

Introduction: An outbreak of Lassa Fever (LF) reported and confirmed in Ondo state, Southwest Nigeria in January 2016 was investigated. This paper provides the epidemiology of the LF and lessons learnt from the investigation of the outbreak.

Methods: The incidence management system (IMS) model was used for the outbreak coordination. Cases and deaths were identified through the routine surveillance system using standard definitions for suspected and confirmed cases and deaths respectively. Blood specimens collected from suspect cases were sent for confirmation at a WHO accredited laboratory. Active case search was intensified, and identified contacts of confirmed cases were followed up for the maximum incubation period of the disease. Other public health responses included infection prevention and control, communication and advocacy as well as case management. Data collected were analysed using SPSS 20, by time, place and persons and important lessons drawn were discussed.

Results: We identified 90 suspected LF cases of which 19 were confirmed by the laboratory. More than half (52.6%) of the confirmed cases were females with majority (73.7%) in the age group ≥ 15 years. The Case Fatality Rate (CFR) of 63.2% among the laboratory-confirmed positive cases where 9 of 19 cases died, was significantly higher compared to the laboratory confirmed negative cases where 6 of the 65 cases died ( CFR; 8.5%) p ≤ 0.05. Two hundred and eighty-seven contacts of the confirmed cases were identified, out of which 267(93.0%) completed  the follow-up without developing any symptoms and 2 (0.7%) developed symptoms consistent with LF and were confirmed by the laboratory. More than half of the contacts were females (64.5%) with most of them (89.2%) in the age group ≥ 25 years.

Discussion: One key lesson learnt from the investigation was that the confirmed cases were mainly primary cases; hence the needs to focus on measures of breaking the chain of transmission in the animal-man interphase during Lassa fever epidemic preparedness and response. In addition, the high case fatality rate despite early reporting and investigation suggested the need for a review of the case management policy and structure in the State. Key Words: Lassa fever, Outbreak Response, Incident Management System, Nigeria

## Introduction

Lassa fever (LF) is a severe acute viral hemorrhagic illness caused by a virus belonging to the family Arenaviridae.^1^^,^^2^ The disease was first discovered in Sierra Leone in the 1950s, but the aetiological agent was first isolated after an outbreak of the disease in a village called Lassa in Borno State, Nigeria claiming the lives of two foreign missionary nurses in 1969.^3^

The virus exhibits persistent, asymptomatic infection, with profuse urinary virus excretion in *Mastomys natalensis* rodents, which serve as the natural reservoir.^1^^,^^2^ The virus is shed in their excreta (urine and faeces) of the rodent which can be aerosolized and inhaled by humans.^4^ Primary mode of spread is from rodent to man through contact with rodent excreta or urine in food or during hunting and processing of rats for consumption. The virus can spread from person-to-person, either within households during care for sick relatives or in health care settings.^5^

The disease, LF, is endemic in West Africa with several outbreaks recorded over the years. Outbreaks of the disease have been in reported in Sierra Leone, Guinea, Liberia, Nigeria, Ghana, and Ivory Coast, Senegal and Mali.^6^ The number of infections per annum has been estimated at 100,000 to 300,000 with approximately 5,000 deaths.^6^^,^^7^^,^^8^^,^^9^^,^^10^ Since the identification of the virus in Nigeria in 1969, yearly outbreaks have been reported in parts of the country,^11^^,^^12^ and more recently in some states including Ondo States.^13^^,^^14^^,^^15^^,^^16^

Despite the yearly outbreaks of LF reported in the country, few literatures exist on the detailed epidemiological investigation and coordinated public health responses used for the control of these outbreaks, especially with modern approaches such as the Emergency Operation Center (EOC) model and most importantly, documented lessons learnt for improved future outbreak responses.

Between December 2015 and February 2016, Nigeria reported suspected cases of LF cases from about 23 states, including Ondo State with preliminary epidemiological and laboratory investigation confirming an outbreak of the disease. The need to document key lessons learnt from the outbreak investigation in Ondo State, in order to improve future responses, incentivises this report. This paper, therefore, describe the epidemiology of the LF outbreak in the State, outline public health responses conducted and document some key lessons learnt to enhance outbreak response.

## Methods

**Outbreak setting**

Nigeria is the most populous country in Africa, with an estimated population of over 160 million (Samson, 2014) and a growth rate of 3.8% per annum. Nigeria has six regional zones with varying ecologies, climates and population characteristics. The zones are divided into 36 states and the federal capital territory, which is further divided into 774 LGAs or districts and 8 812 administrative wards.^17^ Ondo State is one of the 36 states in the Federal Republic of Nigeria situated between longitudes 40 151 E and 60 001E of the Greenwich meridian and latitudes 50 451N and 70 451 N, which are to the North of the equator, in the Southwestern geopolitical zone of the country.^17^

The State has 18 LGAs with three senatorial districts; Ondo North, Central and South and a 2015 projected total population of about 4,489,756 based on the 2006 population census.^18^ The outbreak was restricted to eight LGAs in the north and central senatorial districts of the state. The climate of the areas is highly favoured for the agrarian activities and crops such as cocoa, kola nut, palm tree and arable crops like maize and tubers such as yam and cassava are grown annually.^19^ The annual rainfall is between 1000mm and 1500mm with a high daily temperature of about 300C. The vast majority of the population consists of peasant farmers cultivating food and cash crops at a small-scale level. Livestock keeping is a minor occupation of the population of Ondo State who rear goats, sheep and also do some fish farming. Other economic activities in the state include trading and civil service.^20^

**Field Investigations and public health response**

Following an alert from the disease surveillance and notification officer of two suspected cases of LF at the State Specialist Hospital, Akure on 8 January 2015, the Ondo State Ministry of Health (SMoH) through her Epidemic Management Committee and the World Health Organization (WHO) Ondo State field office reactivated the existing Incident Management System (IMS) at the SMoH, Akure modeled after the Emergency Operating Centre (EOC) for the control of Ebola Virus Disease (EVD) in Nigeria.^21^ The Incident Management System (Appendix I) has previously been used in the investigation of methanol poisoning in the state in 2015.^22^ Strategic groups, which included Epidemiology and Surveillance committee, Case management committee, Infection Control and Burial committee as well as Advocacy and Communication committee were inaugurated and served as the implementation units of the State outbreak response plan for the control the outbreak (Appendix I) .

**Coordination**

The investigation of the outbreak as well as implementation of the outbreak response plan was coordinated by the SMoH officials with technical support provided by the WHO Ondo State field office and country office with the state epidemiologist designated as the incident manager. Disease surveillance and notification officers and volunteer contact tracing personnel recruited were trained by WHO Ondo State field office. Daily coordination meetings were held with situation reports of each strategic group presented and reviewed, and action points developed to strengthen ongoing response activities.

**Surveillance and contact tracing**

The Epidemiology and surveillance subgroup adopted the WHO standard case definition for suspected and confirmed LF cases and deaths to develop an operational case definition for case and death identification during active case search visits to health facilities and communities. The definition was any illness or death following a gradual onset and one or more of the following symptoms: malaise, fever, headache, sore throat, cough, nausea, vomiting, diarrhoea, myalgia, chest pain, hearing loss and a history of contact with excreta of rodents or with a case of LF from December 1, 2015 to April 30, 2016. Also, the name of case, age, sex, address, date of onset of illness, clinical symptoms of each suspected case or death identified were collected using the national LF case investigation form and Integrated Disease Surveillance and Response (IDSR) line-listing forms. Likewise, for any confirmed case or death of LF, contacts tracing and identification were carried out using the WHO definition for a contact, which was, “any person without any disease signs and symptoms but had physical contact which includes sharing the same room/bed, caring for a patient, touching body fluids, or closely participating in a burial with a case (alive or dead) or the body fluids of a case within the last three weeks from the onset of symptoms, within the hospital or community”. The identified contacts were enrolled into 21-day surveillance and follow-up exercise using the national viral haemorrhagic fever contact tracing line list and follow up guidelines and data tools. For each contact under surveillance, daily temperature monitoring and clinical evaluation were carried out for the period of follow up. Moreover, active case searches for additional cases (alive/dead) and retrospective review of hospital records were done in public and private hospitals in the LGAs.

**Laboratory investigation and confirmation**

Laboratory confirmation was performed at the Institute of Lassa Fever Research and Control, Irrua Specialist Teaching Hospital, Edo State, Nigeria, a national reference laboratory for LF diagnosis, treatment and research in Nigeria. The confirmation was based on a positive test using Lassa virus specific reverse-transcriptase PCR (RT-PCR). Laboratory samples (8mls of serum) were collected from each suspected case identified during the investigation. These are packaged in triplicate and transported in the reverse cold chain system by trained laboratory personnel to the laboratory. Feedbacks were provided to the state within 48-72 hours and in some instances more than 72 hours of receipt of specimens, due to unavailability of laboratory diagnostic materials.

**Community/Social mobilization and health education**

Information, Education and Communication (IEC) materials on LF were disseminated to the general public through various channels – print and electronic media, television adverts and radio, posters and banners (Appendix II). The main content of the messages was the discouragement of eating rats and poorly stored/rat-infested foods, symptoms of Lassa fever, and routes of transmission. Community and religious leaders were mobilized and empowered to inform and educate their members. Information on safe referral practices to the nearest hospital was also disseminated. Furthermore, the advocacy-communication subgroup in collaboration with the epidemiology and surveillance subgroup, trained health-care workers across hospitals in the state on case identification using the case definition, universal infectious disease precautionary measures in health care setting and on the importance of intensifying surveillance for additional cases.

**Infection prevention & control**

Procurement and distribution of infection and prevention control (IPC) supplies such as gloves, apron, alcohol, soap, chlorine, including complete Personal Protection Equipment (PPE) kit and IPC Standard Operation procedure (SOPs) guidelines to hospitals were carried out by the Infection prevention and control subgroup. Health workers exposed to confirmed LF cases were given 500mg of ribavirin on a 6 hourly basis for 5 days as PEP prescription. Decontamination exercises using chlorine and disinfectant kit were carried out daily in hospitals and vehicles used for transporting confirmed cases.

**Case management**

Suspected cases with severe clinical presentation while awaiting laboratory results and laboratory-confirmed cases were referred to Irrua Specialist Teaching Hospital in Edo State (about 158km away from Akure, the state capital). The hospital is one of the health institutions designated by the FMoH for LF case diagnosis, treatment and research in the country. Transportation of referred cases was carried out by the case management subgroup in collaboration with infection prevention and control subgroup in ambulances with drivers and personnel who have been trained on IPC.

## Results

Ninety (90) suspected cases of LF from 8 LGAs (Owo, Akure South, Akoko North East, Akoko South East, Akoko South West, Akure North, Ondo West and Owo) of the 18 LGAs in the State were identified (Fig. 1). Of these cases, 19 were confirmed cases by the laboratory (Table 1). The confirmed cases were mainly from 3 LGAs (Akure South, Owo and Ose). The age group mostly affected among the confirmed cases were ≥ 15 years (73.7%) compared to the non-confirmed cases (85.9%), p=0.107) (Table 1). Case Fatality Rate (CFR) among the confirmed cases was 63.2% compared to 8.5% among non-confirmed cases (Table 1).

**Map showing the distribution of suspected Lassa fever cases by geographical location (LGAs) in Ondo State December, 2015 to April 30, 2016:**
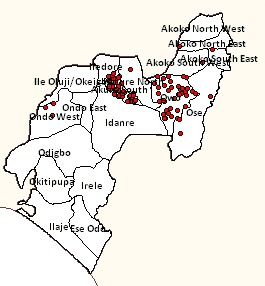

**Table:** Table 1: Characteristics of suspected Lassa fever cases in Ondo State December to April 30, 2016

	Outcome of laboratory investigation		
Variable	Positive for Lassa fever Igm antigen, N=19 (21.1%)	Negative for Lassa fever Igm antigen, N=71 (78.9%)	p-Value
Age group (in years)			
<5	4 (21.1)	4 (5.6)	0.107*
5-14	1 (5.3)	6 (8.5)	
≥ 15	14 (73.7)	61 (85.9)	
Mean age	29.3±1.9	33.6±1.8	
Sex			
Male	9 (47.4)	36 (50.7)	0.796
Female	10 (52.6)	35 (49.3)	
Location (LGA)			
Akure South	8 (42.1)	33 (46.5)	0.698*
Owo	10 (52.6)	28 (39.4)	
Ose	1 (5.3)	2 (2.8)	
Ondo West	0 (0.0)	3 (4.2)	
Akure North	0 (0.0)	2 (2.8)	
Akoko South East	0 (0.0)	1 (1.4)	
Akoko South West	0 (0.0)	1 (1.4)	
Akoko North East	0 (0.0)	1 (1.4)	
Outcome of case management			
Alive	7 (36.8)	65 (91.5)	<0.001*+
Dead	12 (63.2)	6 (8.5)	
Median days between onset of symptom and presentation at the	2.0 (0 -17) days	2.0 (0 -23) days	
Exposure to a confirmed case			
Yes	2 (10.5)	7 (10.0)	0.932
No/Unknown	17 (89.5)	64 (90.0)	

*Fisher Exact Test, +Significant at p ≤ 0.05

Fig. 2 shows the epidemic curve of the outbreak, which began in the epidemiological week 53 of 2015 (28th December, 2015 to 3rd January 2016) with the index cases reported and confirmed in epidemiological week 1 of 2016 (4th to 10th January, 2016). Afterwards, there was a steady increase in the number of cases between epidemiological week 1 of 2016 (4th to 10th January, 2016) and epidemiological week 5 of 2016 (1st to 7th February 2016) with the outbreak reaching its peak in epidemiological week 4 (25th to 31st January, 2016) and week 5 (1st to 7th February, 2016) of 2016. Thereafter, fluctuations in the number of cases were noted with a steady decline in the cases recorded between epidemiological week 11 (14th to 10th March, 2016) and epidemiological week 14 (4th to 10th April, 2016) of 2016 (Fig. 2).

**Epidemic curve of Lassa fever cases (suspected and confirmed) in Ondo State December 2015 to April 2016:**
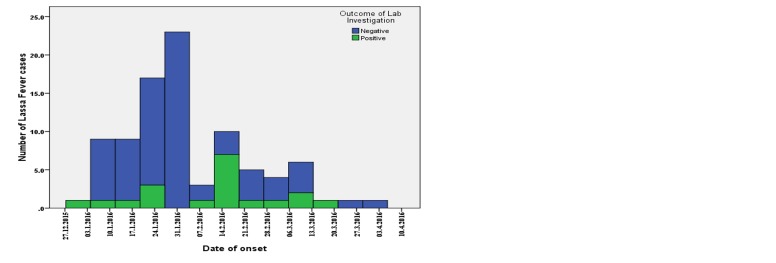

Furthermore, 287 contacts of the confirmed cases were identified and followed-up as shown in Table 2 and Fig. 3. More than half of these contacts were females (64.5%) with most of them (89.2%) in the age group ≥ 25 years. Among the contacts, two (0.7%) developed symptoms that were consistent with LF (fever, malaise, sore throat, vomiting, diarrhoea) and were laboratory-confirmed. Of these two cases, one died while the other recovered after treatment (Fig. 4). Two hundred and seventy-six (96.2%) completed the follow-up exercise without developing any symptoms consistent with LF (Fig. 4).

**Table:** Table 2: Characteristics of Lassa fever contacts followed-up from December 2015 to April 2016

Variable	N (%) (n=287)
Age group (years)	
<5	5 (1.7)
5-14	5 (1.7)
15-24	21 (7.3)
≥ 25	256 (89.2)
Gender	
Male	102 (35.5)
Femalef	185 (64.5)
Occupation	
Health care worker	241 (84.0)
Businessman/trader	30 (10.5)
Student	11 (3.8)
Teacher	4 (1.4)
Clergy	1 (0.3)
Type of contact	
Hospital	240 (83.6)
Community	47 (16.4)
Neighbour	16 (5.6)
LGA	
Akure South	144 (50.2)
Owo	125 (43.6)
Ose	14 (4.9)
Akure North	3 (1.0)
Idanre	1 (0.3)

**Relationship of contacts with confirmed cases of Lassa fever in Ondo State, December 2015 to April 2016:**
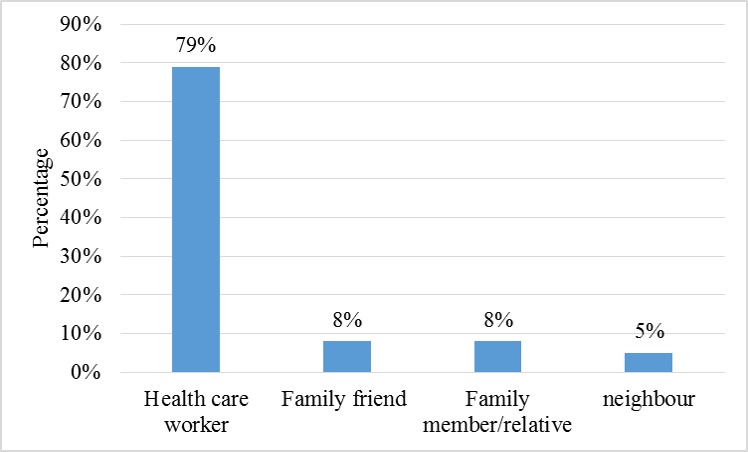

**Outcome of Lassa Fever contact follow-up, December 2015 to April, 2016:**
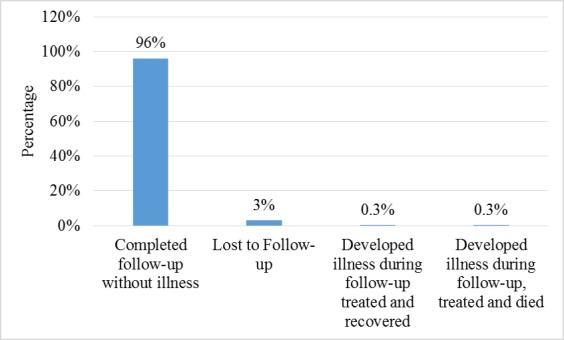

## Discussion

In this investigation, although a considerable number of suspected cases were identified, a few were laboratory confirmed indicating a highly sensitive surveillance system. In addition, more than half of the confirmed cases were female with the majority in the adolescent and the young adult age group. The CFR was high despite early detection and reporting of suspected cases indicating challenges in the case management protocol. Furthermore, contacts tracing and follow-up were successful, to some extent, with only very few contacts developing the disease.

The finding in which the proportion of those with disease was higher among females compared to males is inconsistent with a previous outcome of investigation of LF in Nigeria as reported by Ajayi et.al. 2013.^13^ Differences in exposures between male and female may have been responsible for this finding, given that gender role have not been shown to be a significant factor in the transmission of LF and other VHFs.^23^ Most of the confirmed LF cases in this investigation were in the age group ≥ 15 years. Ajayi et.al, 2013 in a similar investigation of LF outbreak in Ebonyi State Southeast Nigeria reported that almost all the cases recorded during the outbreak were in the older age group of ≥ 20 years.^13^

The high CFR (63.2%) among the laboratory-confirmed cases in this outbreak is slightly lower than those reported in other part of Africa such as Sierra Leone (69%), and however higher than those reported previously in several outbreaks in Nigeria.^24^ For instance, Getso et.al, 2014 reported a CFR of 40% during an outbreak of LF in Taraba state, northern Nigeria in the year 2012.^25^ Similarly, Ajayi et.al, 2013 reported a CFR of 40% during an outbreak of LF in Ebonyi state, south-eastern Nigeria.^13^ The higher CFR reported in our investigation could be attributed partly to the strategy of case management adopted by the case management subgroup where LF cases were referred outside the state for treatment due to the absence of a well equipped infectious disease treatment facility to manage these cases in the state. Furthermore, the quality of care received by the LF cases could have played some roles as the treatment cost at the referral hospital were partly the responsibility of the family members/caregivers of these cases thereby leaving families that were poor with inability to pay for good-quality health care for their wards. However, information on the cost of treatment for LF case and the economic burden on caregiver/families were not captured in our investigation.

The epidemic curve of this outbreak suggests a continuous source pattern of transmission, which is inconsistent with previous outbreaks reported in Nigeria.^13^ Apart from the two contacts who developed the disease, there are no known human sources of the disease for the others. Contrary to what we observed, previous investigations have shown that transmission of the VHFs such as LF has been related to direct contact with blood and other bodily fluids of people who are acutely ill.^23^

The spread of the disease during the outbreak was contained within a short period of time through an effective public health response strategy using the IMS/EOC model. This model uses an inter-sectoral collaborative approach in response to disease and other public health condition outbreaks resulting in coordination and resource mobilization. It relies substantially on surveillance for cases and contacts, case management and infection control, and laboratory diagnosis of cases, as well as effective public information and communication.

Previous studies on disease outbreak control have also reported the use of the EOC model in prompt and successful containment of diseases and other public health condition outbreaks in Nigeria and African regions.^21^^,^^22^^,^^26^ Shuaib et.al, 2014 reported that the use of the EOC model to coordinate the outbreak response and consolidate decision making during the Ebola Virus Disease outbreak in Nigeria in 2014 was largely significant with helping to contain the disease outbreak early in Nigeria.^21^

Similarly, Adeyanju et. al, 2015 in the investigation of the acute methanol poison outbreak in Ondo state, Nigeria, reported the use of the EOC model as an efficient outbreak response model in the coordination and response to outbreaks of public health conditions.^22^ Also, Kouadio et. al, 2016, reported that the use of the EOC model contributed to a high-level implementation of the National Polio Emergency Operational Plans strategies and activities in Nigeria with a resultant reduction in the number of WPV cases from 122 in 2012 to 53 (57% reduction) in 2013; and 6 (90% reduction) in 2014.^26^

The outbreak did not record any known nosocomial transmission of the disease among health-care workers who treated confirmed LF cases prior to their referral. Transmission among health-care workers has been a common occurrence in previous LF outbreaks in Nigeria with high incidence and fatality recorded among health workers providing care for confirmed LF and other VHFs cases.^5^^,^^11^^,^^13^^,^^25^^,^^27^ The high awareness of the disease among health-care workers, with a consequence of minimizing exposure may be partly responsible. The awareness might have created fear to care for LF patients, due to inadequate infra-structural and logistic support to the health facilities. Although several awareness creation and sensitization trainings on infection prevention and control in hospital settings were conducted for health workers across the state, these workers were not provided with PPEs materials and tools to protect themselves in most cases. There were no holding centres or isolation wards for VHF in any facility in the State. The infectious disease centre in the State capital, built to care for VHFs and other infectious diseases was not functional, due to lack of Manpower. The confirmed LF cases were transferred to the specialist hospital outside the State on receipt of the confirmation results, sometimes in an undesirable manner. Health workers who were highly exposed to confirmed LF cases were not provided with oral ribavirin as a post exposure prophylaxis on time, some of them were reported to procure the drugs on their own. We, however, did not obtain data on PEP to evaluate its impact.

Furthermore, the finding that most of the contacts of the LF confirmed cases identified, were females, is consistent with Iroezindu et. al, 2015 who reported similar findings during an outbreak of LF in Enugu southeastern Nigeria.^28^ The cultural practices in Nigeria, where the social burden of caring for the sick falls to the lot of women thereby increasing their vulnerability to infectious diseases may partly explain the observation. In addition, the high proportion of health- care workers identified as contacts for follow-up exercise is consistent with previous investigation,^28^ hence the need to provide adequate infection preventive measures within the health care setting to reduce exposure of health-care workers to infectious diseases.

## Conclusion

Finally, the main limitation of this investigation was our inability to conduct a risk factor survey to identify factors associated with the cause of the outbreak. However, the strong collaboration among partners as well as the incident management approach to the outbreak was a key to the control and containment of the outbreak.

One key lesson learnt from the investigation was that the confirmed cases were mainly primary cases; hence the need to focus on measures of breaking the chain of transmission in the animal-man interphase in LFs epidemic preparedness and response. In addition, the high case fatality rate despite early reporting and investigation suggested the need for a review of the case management policy and structure in the State. There is the need to ensure that holding wards are established in all the health facilities and that trained, and highly motivated manpower are provided at these and the isolation centres at the State Capital and other specialist hospitals across the State.

## Funding

The authors received no specific funding for this work.

## Competing Interests

The authors have declared that no competing interests exist

## Corresponding Author

Elvis E. Isere: Email: elvisisere@gmail.com; Phone number: +23408030480305

## Data Availability

Data are available in Figshare using the following link: https://figshare.com/s/76a75e5a0292267576d3.

## APPENDIX

**Figure:**
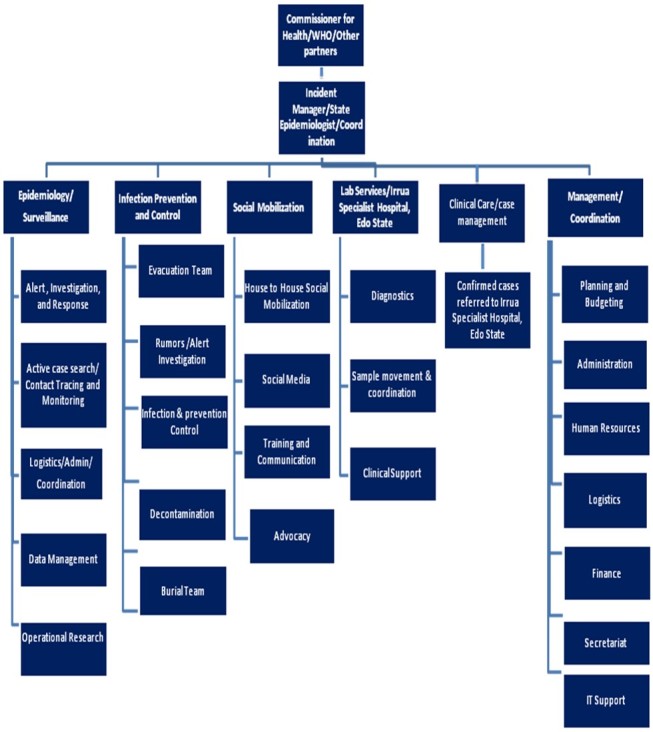
Appendix I: Incident Management system structure used for the investigation of the outbreak and coordinating public health response

**Figure:**
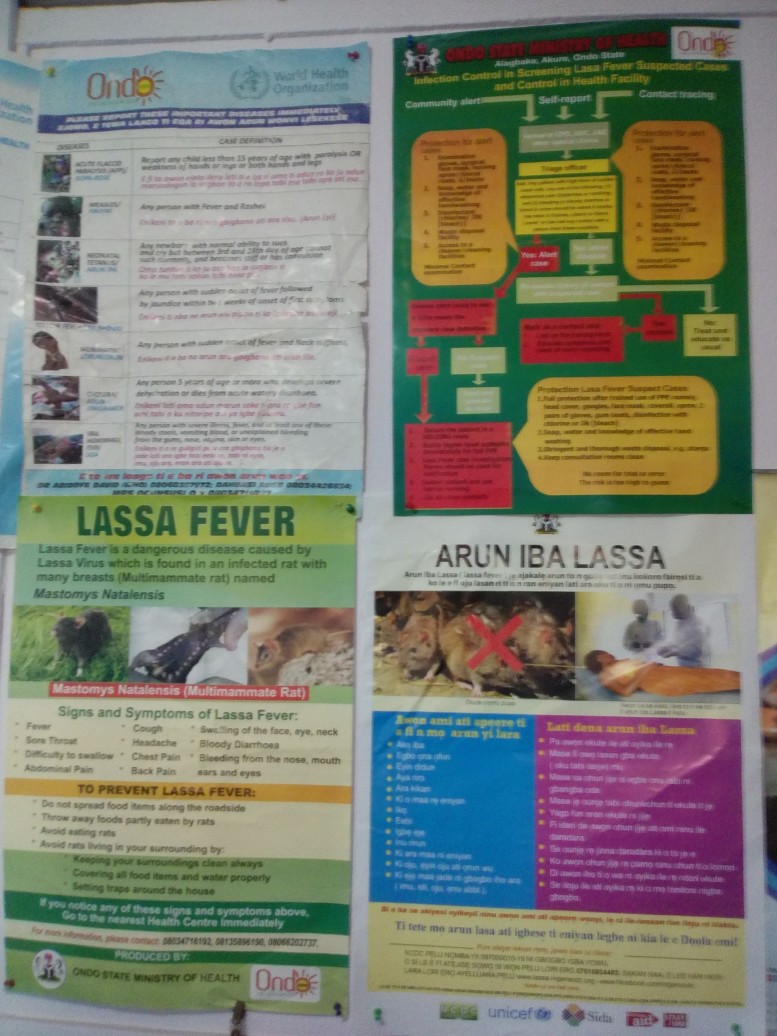
Appendix II: IEC posters produced for sensitization during the outbreak by the SMoH and health development partner

**Figure:**
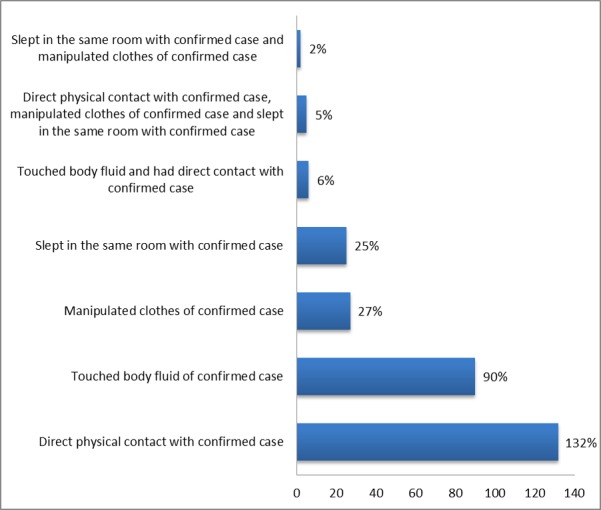
Appendix III: Source of exposure of contacts to confirmed case of Lassa fever in Ondo State, December 2015 to April 2016
